# Impact of maras powder on mandibular bone microarchitecture: a fractal and radiomorphometric study

**DOI:** 10.1186/s12880-026-02360-w

**Published:** 2026-05-02

**Authors:** Emine Ararat, Ayşe Gül Öner Talmaç

**Affiliations:** Department of Dentomaxillofacial Radiology, Faculty of Dentistry, Kahramanmaras Sutcu Imam University, Kahramanmaras, Turkey

**Keywords:** Fractals, Mandible, Maras powder, Panoramic, Radiograph, Smoking

## Abstract

**Background:**

The aim of this study was to examine how maras powder (MP) affects on the cortical and trabecular bone of the mandible using the radiomorphometric indexes and fractal dimension (FD).

**Methods:**

A retrospective analysis of radiographic records of 150 male individuals, 50 of whom used MP, 50 of whom smoked cigarettes, and 50 of whom were healthy and did not use any tobacco derivatives, was performed. Cortical bone was evaluated with mandibular cortical width (MCW) and panoramic mandibular index (PMI). Trabecular bone in mandibular anterior was evaluated by FD. The ANOVA test was used to compare normally distributed variables across the three groups, and the Kruskal Wallis test was used to compare non-normally distributed variables across the three groups.

**Results:**

The mean age of MP users was 42.92 ± 10.21; in smokers, 40.46 ± 10.51; and in the healthy control group, 40 ± 15.05. When the FD measurements were examined in regions of interest (ROI) 1, ROI 2, ROI 3, and the mean ROI values, no significant difference was found between the three groups in terms of FD (*p* > 0.05), but the fractal dimension was found to be lower in individuals using MP. No significant difference was found between the groups in terms of histogram values ​​and MCW and PMI measurements (*p* > 0.05).

**Conclusion:**

No significant differences were found between users of MP, smokers, and healthy individuals. However, the decreasing trend in FD values ​​may indicate early effects of MP. Studies with larger sample sizes and advanced imaging techniques are needed.

## Introduction

Tobacco use is rapidly increasing in developing countries. While tobacco consumption is primarily in the form of cigarettes, other forms of smokeless tobacco use are also quite common. Available under various names and with varying characteristics in different countries, primarily in Southeast Asia, smokeless tobacco is used by many cultures around the World [1–3]. The Maras Powder (MP) is a smokeless tobacco product widely used in Türkiye, especially in Southeastern Anatolia and the Mediterranean regions [4]. After drying and pulverizing tobacco leaves, they are mixed with ashes from vine, oak, or walnut sticks in a ratio of 1/2 to 1/3 and then slightly moistened with water before being used orally. The prepared mixture is then wrapped directly in tobacco or cigarette paper and placed between the lower lip, sometimes the upper lip, the buccal mucosa, and the chin. It is usually left in the mouth for 5–10 min, sometimes 1–2 h, and then spit out [5]. The nicotine content of MP, obtained from a plant called Nicotiana rustica linn, is 6–10 times higher than Nicotina tobacum, from which cigarettes are produced [6].

It has been noted that panoramic radiographs do not adequately demonstrate changes caused by osteoporosis in the mandible and only provide a rough estimate [7]. However, objective assessments of trabecular structures can be made using numerical results from various computer-assisted analyses in panoramic radiographs. Fractal analysis (FA), a digital method used to analyze complex images by examining their fundamental components, has been widely used in scientific research to analyze biological images in recent years. In addition to FA, radiomorphometric measurements such as panoramic mandibular index (PMI) and mandibular cortical width (MCW) are simple and effective methods for detecting osteoporotic conditions [7–11]. Previous studies have suggested that smokeless tobacco use is a risk factor for osteoporosis [12, 13]. A link has also been found between smoking and osteoporotic fractures [14, 15]. The null hypothesis of this study was that MP use does not affect mandibular bone microarchitecture, and there is no significant difference in FD and radiomorphometric indices between MP users, non-users and smokers.

The purpose of this study was to evaluate and compare FA and radiomorphometric measurements of the mandibular bone in digital panoramic radiography in MP users, smokers, and non-smokers. To our knowledge, no studies in the literature have been identified that have conducted fractal and radiomorphometric analyses of mandibular bone changes in patients using MP. This study is a pioneering study in the field.

## Materials and methods

### Study design

This retrospective study included individuals who visited the Kahramanmaraş Sütçü İmam University Department of Dentomaxillofacial Radiology between 2024 and 2025 years and all individuals who met the inclusion criteria during the study period were included. A power analysis was performed using G*Power software (Version 3.1, Heinrich Heine University, Düsseldorf, Germany). For a one-way ANOVA with three groups, assuming a medium effect size (f = 0.25), a significance level of 0.05, and a total sample size of 150 participants (50 per group), the statistical power of the study was calculated as 0.84.

Ethical approval for the study was obtained from the Medical Research Ethics Committee of Kahramanmaraş Sütçü İmam University (Approval No:2025/26 − 02), and all procedures were conducted in accordance with the Declaration of Helsinki. Written informed consent was obtained from all participants prior to inclusion.

### Patient selection

All participants whose medical histories and clinical data were recorded were male, and the average ages of the groups were similar. Based on this datas, participants were categorized into three groups: individuals who used MP, cigarette smokers, and healthy controls with no tobacco exposure. Individuals in the MP group were included only if they had a history of regular use for at least 5 years. In addition, all users reported habitual placement of MP in the mandibular anterior buccal region, ensuring consistent local exposure. The smoking group had been using various tobacco products for at least 5 years. Individuals using MP repeated this practice at least three times a day. The group of smokers consisted of patients who smoked one pack or more of cigarettes a day. All individuals in the three groups included in the study had no systemic or metabolic diseases affecting the jawbone (osteoporosis, diabetes, etc.) and no ongoing medication use (corticosteroids, bisphosphonates, etc.). They also had no alcohol use or hormonal disorders. Subjects with systemic or metabolic diseases, as well as those presenting with any cystic, tumoral, dysplastic, or other pathological lesions in the jaws, were excluded. Panoramic images exhibiting motion, positioning errors, or technical artifacts were also omitted from the analysis.

### Radiographic measurements

**Fig. 1:**
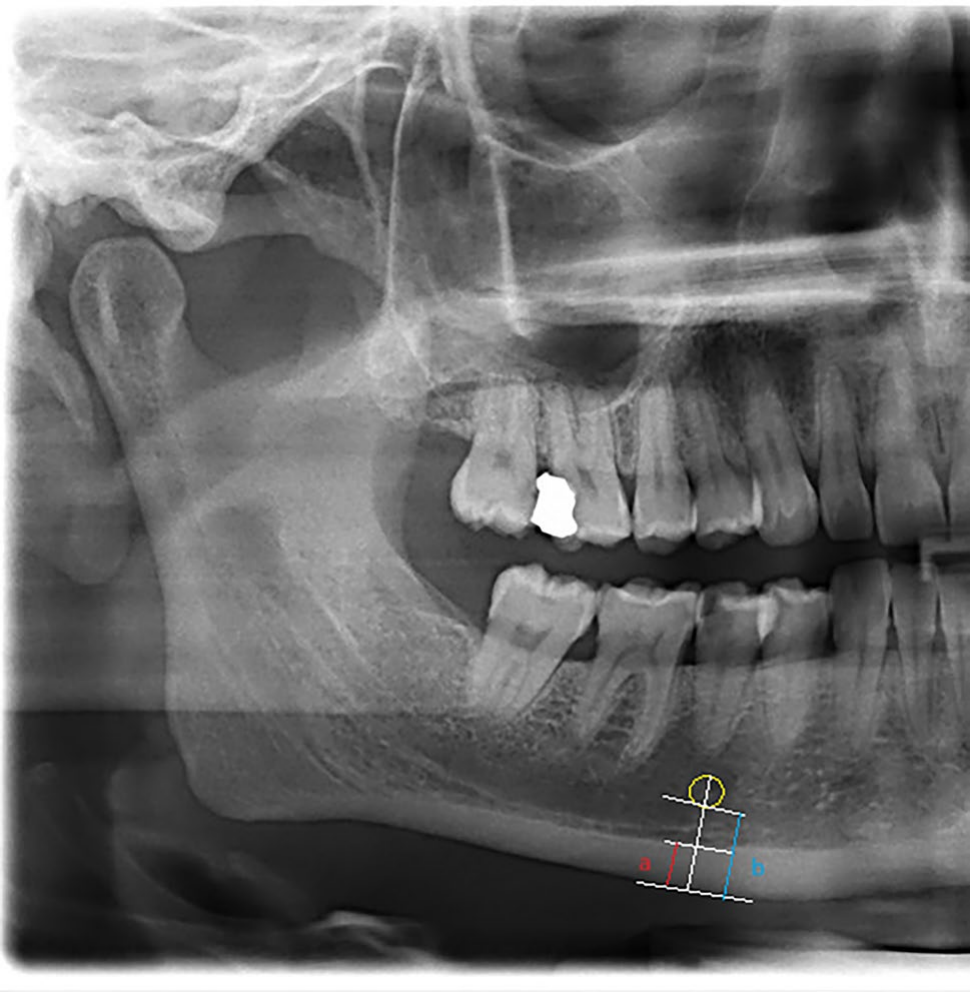
Measurement of radiomorphometric indices. mandibular cortical thickness (a) and panoramic mandibular index (ratio a/b). The red circle outlines the mental foramen

All radiographic measurements (MCW and PMI) were performed by a single experienced oral and maxillofacial radiologist to ensure consistency. To assess intra-observer reliability, 30 randomly selected images (approximately 20% of the total sample) were re-evaluated after a two-week interval under the same conditions. Intra-observer agreement was assessed using the intraclass correlation coefficient (ICC) based on a two-way mixed-effects model with absolute agreement. Quantitative evaluation of the mandibular bone was performed using MCW and PMI parameters. MCW was determined by measuring the perpendicular distance between two parallel tangents one along the superior border and the other along the inferior border of the mandibular cortex at the mental foramen level (line a). The distance from the lower margin of the mental foramen to the inferior border of the mandible was recorded as line b. The PMI value was calculated by dividing cortical thickness by this vertical distance (a/b) (Fig. 1) [16].

### Fractal analysis

FD analysis was performed by a single experienced oral and maxillofacial radiologist to ensure consistency in regions of interest (ROI) selection and image processing procedures. To evaluate measurement reliability, intra-observer agreement was assessed by repeating the analysis on 30 randomly selected images after a two-week interval. Intra-observer reliability was assessed using the ICC.

**Fig. 2:**
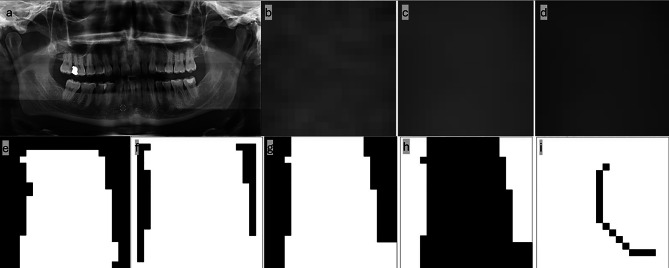
Fractal analysis stages; (**a**) determination of ROI area, (**b**) cropped and duplicated version of the image, (**c**) applying gaussian filter, (**d**) subraction process, (**e**) make binary application, (**f**) erode, (**g**) dilate, (**h**) invert, (**f**) Skeletonize

Since MP is used in the mandibular anterior region, FA was performed in three defined ROI in the anterior mandibular. The first ROI (20 × 20 pixels) was located between the apices of the right canine and lateral incisor, the second between the anterior canine and lateral incisor, and the third between the left canine and lateral incisor (Fig. 2). The mandibular cortical boundary, the mandibular canal, and the periodontal ligament space were consistently excluded from all ROIs.

The procedure followed the box-counting method originally described by White and Rudolph [17]. Each selected area from the panoramic radiograph was cropped and duplicated. A Gaussian blur filter (σ = 35 pixels) was applied to the duplicated image to reduce intensity fluctuations due to variations in bone thickness and overlying soft tissue. The blurred image was subtracted from the original, and a grayscale offset of 128 was added to enhance the contrast between trabecular and marrow spaces. The resulting image was binarized using the “Threshold” tool, noise was minimized via the “Erode” function, and the trabecular borders were accentuated using the “Dilate” command. The image was then inverted, and the trabecular framework was skeletonized using the “Skeletonize” tool.

**Fig. 3:**
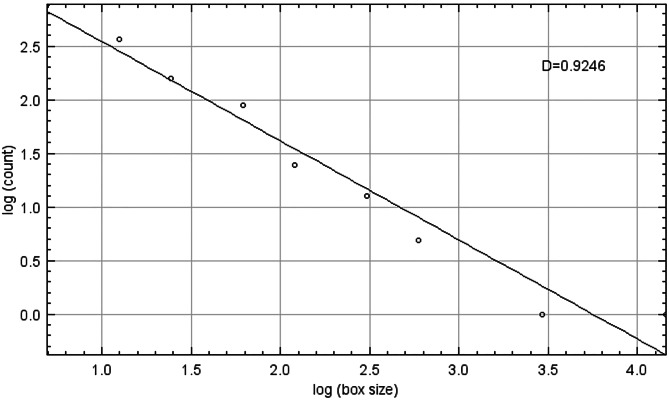
Graph showing fractal analysis value

FD values were obtained using the **“**Fractal Box Counter**”** plugin. The image was divided into boxes of 2, 3, 4, 6, 8, 12, 16, 32, and 64 pixels, and the number of boxes containing trabecular structures was calculated for each size. The logarithmic relationship between box size and count was plotted, and the slope of the regression line represented the FD value (Fig. 3).

### Histogram analysis

**Fig. 4:**
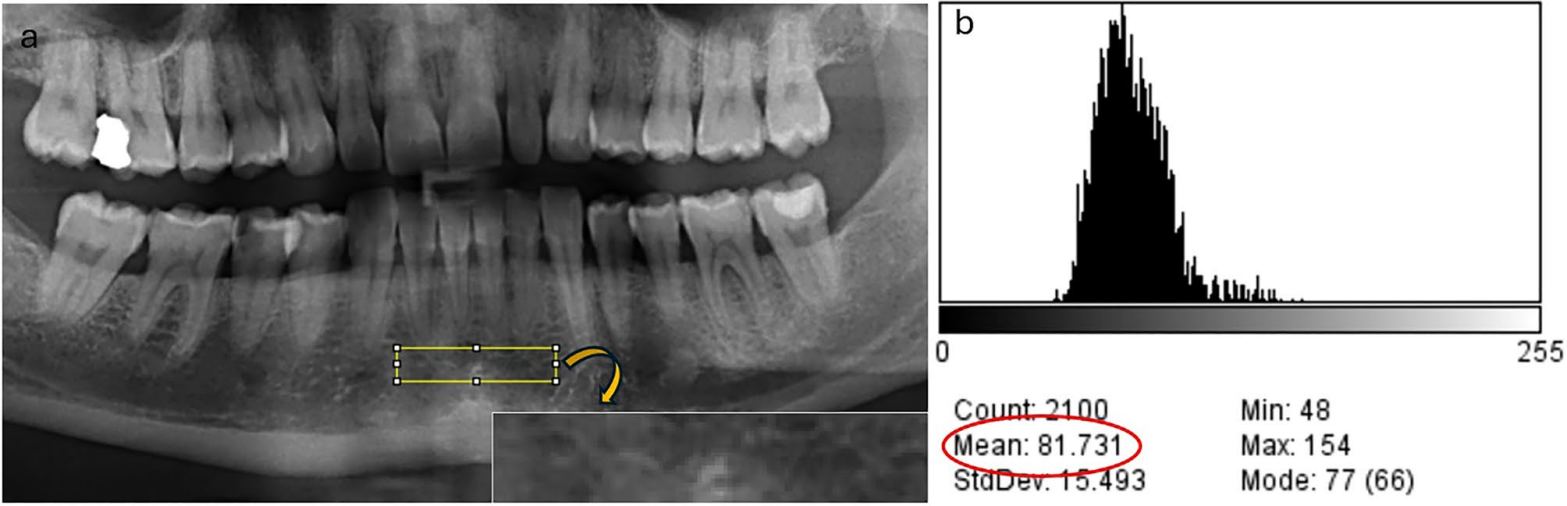
Histogram analysis; (**a**) determination of ROI area, (**b**) mean value

Digital panoramic images are composed of pixels, each representing a grayscale intensity corresponding to local brightness or density. Histogram analysis (HA) was performed to numerically assess pixel intensity distribution. A rectangular ROI (100 × 20 pixels) was selected between the mandibular canines using ImageJ software. The selected area was duplicated, and the mean grayscale intensity was obtained using the histogram function (Fig. 4).

### Statistical analysis

The Shapiro–Wilk test was applied to assess the normality of data distribution. For variables with normal distribution, one-way ANOVA was used to compare differences among the three groups. For non-normally distributed variables, the Kruskal–Wallis test was applied.

Associations between categorical variables were evaluated using the chi-square test, and correlations between numerical variables were examined using the Spearman rank correlation coefficient. All statistical analyses were conducted using SPSS version 22.0 (IBM Corp., Armonk, NY, USA). A p-value of < 0.05 was considered statistically significant.

## Results

The intra-observer reliability analysis demonstrated excellent agreement for all measurements. The ICC values were as follows: FD (ICC = 0.92), MCW (ICC = 0.90), and PMI (ICC = 0.95), indicating high consistency of repeated measurements.

The mean age of MP users was 42.92 ± 10.21 years; 40.46 ± 10.51 years in smokers; and 40 ± 15.05 years in the healthy control group. No significant difference was found between the groups in terms of age (*p* = 0.483). All individuals in the study groups were male. When examining the FA measurements for ROI 1, ROI 2, and ROI 3, and the mean ROI values, no significant difference was found in fractal dimension between the three groups (*p* > 0.05). There were also no significant differences between the groups in terms of histogram values, MCW and PMI measurements (*p* > 0.05) (Table 1).

**Table 1:** Comparison of FD, histogram, MCW, PMI measurements between study groups

	Group of using Maraş powder		Smokers group		control group		
	Mean.± SD	Medyan (%25-%75)	Mean.± SD	Medyan (%25-%75)	Mean.± SD	Medyan (%25-%75)	P
ROI 1 FD	0,78 ± 0,14	0,8 (0,68 -0,85 )	0,82 ± 0,11	0,82 (0,76 -0,87 )	0,84 ± 0,12	0,85 (0,77 -0,92 )	0,285†
ROI 2 FD	0,75 ± 0,23	0,82 (0,69 -0,88 )	0,84 ± 0,12	0,82 (0,77 -0,92 )	0,79 ± 0,16	0,83 (0,74 -0,88 )	0,673ỻ
ROI 3 FD	0,83 ± 0,11	0,82 (0,77 -0,89 )	0,83 ± 0,1	0,85 (0,76 -0,88 )	0,86 ± 0,12	0,88 (0,81 -0,93 )	0,569†
FD (mean)	0,79 ± 0,1	0,82 (0,73 -0,87 )	0,83 ± 0,07	0,82 (0,79 -0,87 )	0,83 ± 0,08	0,84 (0,8 -0,88 )	0,448ỻ
Histogram	107,63 ± 24,12	100,99 (91,58 -125,77 )	96,29 ± 19,19	97,41 (83,87 -105,95 )	102,09 ± 23,09	98,19 (88,4 -113,37 )	0,217†
MCW (a)	19,35 ± 3,02	19,68 (18,04 -21,97 )	19,12 ± 4,28	18,38 (16,41 -21,04 )	20,04 ± 3,44	19,62 (17,11 -22,64 )	0,659†
(b)	48,04 ± 8,91	46,46 (42,61 -55,69 )	48,83 ± 7,15	48,05 (42,72 -53,51 )	48,12 ± 6,45	46,51 (43,65 -51,54 )	0,924†
PMI (a/b)	0,41 ± 0,09	0,42 (0,35 -0,46 )	0,39 ± 0,08	0,38 (0,33 -0,43 )	0,43 ± 0,1	0,39 (0,35 -0,5 )	0,475†

As observed in Table 2, a strong correlation was found between the mean fractal dimension and ROI 1 and ROI 2 in MP users. Furthermore, a strong negative correlation was observed between the radiomorphometric indices PMI and age in MP users.

**Table 2:** Correlation between measurements in Maraş powder users

		Age	ROI 1	ROI 2	ROI 3	FD (mean)	Histogram	MCW (a)	b	PMI (a/b)
Age	**r**	1,000	,139	,024	-,241	-,037	-,177	-,075	,440^*^	-,528^**^
	**P**	.	,516	,910	,257	,865	,407	,728	,032	,008
	**N**	50	50	50	50	50	50	50	50	50
ROI 1	**r**	,139	1,000	,294	-,356	,600^**^	-,190	-,119	-,068	,083
	**P**	,516	.	,164	,088	,002	,373	,581	,751	,701
	**N**	50	50	50	50	50	50	50	50	50
ROI 2	**r**	,024	,294	1,000	-,028	,749^**^	-,090	,027	-,066	,118
	**P**	,910	,164	.	,896	,000	,677	,899	,759	,583
	**N**	50	50	50	50	50	50	50	50	50
ROI 3	**r**	-,241	-,356	-,028	1,000	,240	,005	,104	,269	-,263
	**P**	,257	,088	,896	.	,259	,981	,627	,204	,215
	**N**	50	50	50	50	50	50	50	50	50
FD mean	**r**	-,037	,600^**^	,749^**^	,240	1,000	-,239	-,024	,077	-,034
	**P**	,865	,002	,000	,259	.	,260	,910	,722	,873
	**N**	50	50	50	50	50	50	50	50	50
Histogram	**r**	-,177	-,190	-,090	,005	-,239	1,000	-,102	-,221	,091
	**P**	,407	,373	,677	,981	,260	.	,636	,300	,673
	**N**	50	50	50	50	50	50	50	50	50
MCW (a)	**r**	-,075	-,119	,027	,104	-,024	-,102	1,000	,420^*^	,329
	**P**	,728	,581	,899	,627	,910	,636	.	,041	,117
	**N**	50	50	50	50	50	50	50	50	50
b	**r**	,440^*^	-,068	-,066	,269	,077	-,221	,420^*^	1,000	-,646^**^
	**P**	,032	,751	,759	,204	,722	,300	,041	.	,001
	**N**	50	50	50	50	50	50	50	50	50
PMI (a/b)	**r**	-,528^**^	,083	,118	-,263	-,034	,091	,329	-,646^**^	1,000
	**P**	,008	,701	,583	,215	,873	,673	,117	,001	.
	**N**	50	50	50	50	50	50	50	50	50

As observed in Table 3, a moderate correlation was observed between histogram measurements and age in smokers. A strong correlation was also found between the fractal dimension mean and ROI 3 in smokers. A moderately significant correlation was observed between the radiomorphometric indices MCW and ROI 2, while a strong positive correlation was observed between MCW and PMI.

**Table 3:** Correlation between measurements in smokers

		Age	ROI 1	ROI 2	ROI 3	FD mean	histogram	MCW (a)	b	PMI (a/b)
Age	r	1,000	-,023	-,145	-,210	-,239	,473^*^	-,280	-,227	-,062
	P	.	,916	,498	,325	,261	,020	,185	,285	,775
	N	50	50	50	50	50	50	50	50	50
ROI 1	r	-,023	1,000	-,140	,499^*^	,693^**^	,277	-,071	,436^*^	-,466^*^
	P	,916	.	,514	,013	,000	,189	,740	,033	,022
	N	50	50	50	50	50	50	50	50	50
ROI 2	r	-,145	-,140	1,000	-,130	,309	,100	,652^**^	,246	,500^*^
	P	,498	,514	.	,546	,142	,642	,001	,247	,013
	N	50	50	50	50	50	50	50	50	50
ROI 3	r	-,210	,499^*^	-,130	1,000	,721^**^	,090	,163	,534^**^	-,237
	P	,325	,013	,546	.	,000	,677	,446	,007	,265
	N	50	50	50	50	50	50	50	50	50
FD mean	r	-,239	,693^**^	,309	,721^**^	1,000	,277	,429^*^	,700^**^	-,070
	P	,261	,000	,142	,000	.	,189	,037	,000	,744
	N	50	50	50	50	50	50	50	50	50
Histogram	r	,473^*^	,277	,100	,090	,277	1,000	,164	,187	,192
	P	,020	,189	,642	,677	,189	.	,443	,381	,369
	N	50	50	50	50	50	50	50	50	50
MCW (a)	r	-,280	-,071	,652^**^	,163	,429^*^	,164	1,000	,529^**^	,703^**^
	P	,185	,740	,001	,446	,037	,443	.	,008	,000
	N	50	50	50	50	50	50	50	50	50
b	r	-,227	,436^*^	,246	,534^**^	,700^**^	,187	,529^**^	1,000	-,173
	P	,285	,033	,247	,007	,000	,381	,008	.	,418
	N	50	50	50	50	50	50	50	50	50
PMI (a/b)	r	-,062	-,466^*^	,500^*^	-,237	-,070	,192	,703^**^	-,173	1,000
	P	,775	,022	,013	,265	,744	,369	,000	,418	.
	N	50	50	50	50	50	50	50	50	50

When the numerical parameters of the control group were examined, a very strong correlation was found between MCW and PMI values. ROI mean was strongly correlated with ROI 1 and ROI 2 (Table 4).

**Table 4:** Correlation between measurements in the control group

	Group of using Maraş powder		Smokers group		Control group		
	Mean.± SD	Medyan (%25-%75)	Mean.± SD	Medyan (%25-%75)	Mean.± SD	Medyan (%25-%75)	*P*
ROI 1 FD	0,78 ± 0,14	0,8 (0,68 − 0,85 )	0,82 ± 0,11	0,82 (0,76 − 0,87 )	0,84 ± 0,12	0,85 (0,77 − 0,92 )	0,285†
ROI 2 FD	0,75 ± 0,23	0,82 (0,69 − 0,88 )	0,84 ± 0,12	0,82 (0,77 − 0,92 )	0,79 ± 0,16	0,83 (0,74 − 0,88 )	0,673ỻ
ROI 3 FD	0,83 ± 0,11	0,82 (0,77 − 0,89 )	0,83 ± 0,1	0,85 (0,76 − 0,88 )	0,86 ± 0,12	0,88 (0,81 − 0,93 )	0,569†
FD (mean)	0,79 ± 0,1	0,82 (0,73 − 0,87 )	0,83 ± 0,07	0,82 (0,79 − 0,87 )	0,83 ± 0,08	0,84 (0,8 − 0,88 )	0,448ỻ
Histogram	107,63 ± 24,12	100,99 (91,58–125,77 )	96,29 ± 19,19	97,41 (83,87–105,95 )	102,09 ± 23,09	98,19 (88,4 -113,37 )	0,217†
MCW (a)	19,35 ± 3,02	19,68 (18,04–21,97 )	19,12 ± 4,28	18,38 (16,41 − 21,04 )	20,04 ± 3,44	19,62 (17,11–22,64 )	0,659†
(b)	48,04 ± 8,91	46,46 (42,61 − 55,69 )	48,83 ± 7,15	48,05 (42,72 − 53,51 )	48,12 ± 6,45	46,51 (43,65 − 51,54 )	0,924†
PMI (a/b)	0,41 ± 0,09	0,42 (0,35 − 0,46 )	0,39 ± 0,08	0,38 (0,33 − 0,43 )	0,43 ± 0,1	0,39 (0,35 − 0,5 )	0,475†

## Discussion

The present study aimed to evaluate the effects of MP and cigarette use on mandibular bone microarchitecture using fractal analysis and radiomorphometric indices. The findings revealed no significant differences among MP users, cigarette smokers, and non-users in terms of FD values obtained from different ROIs, as well as histogram parameters, MCW, and PMI. However, significant correlations were observed between FD values and radiomorphometric indices within each group, suggesting an association between trabecular bone complexity and cortical bone characteristics. These findings indicate that although tobacco exposure may influence bone structure, such effects may not be sufficiently pronounced to produce statistically significant intergroup differences under the conditions of this study.

An important aspect of the present study is the localized pattern of MP use. All individuals in the MP group reported habitual placement of the product in the mandibular anterior buccal region for at least five years. Chronic localized exposure to tobacco and its chemical components may affect the surrounding periodontal tissues and alveolar bone through inflammatory responses, vascular changes, and alterations in bone metabolism parameters. According to the results of this study, although there was no significant difference between the groups, the FD was observed to be reduced in the group using MP.

In dental practice, the status of the supporting bone influences diagnosis, treatment planning, and clinical outcomes. Radiological imaging techniques play a crucial role in assessing changes in bone structure [18]. Panoramic radiographs, with their broad anatomical coverage and low radiation exposure, are widely used in the maxillomandibular region [11]. Non-invasive techniques such as FA on panoramic radiography reveal the microarchitectural structure of trabecular bone and enable the detection of osteoporotic changes in alveolar bone [19]. Low FD values ​​are associated with low bone mineral density; FA of panoramic radiographs is an effective and non-invasive screening method as a surrogate or complementary diagnostic indicator for the diagnosis of osteoporosis [18]. Tobacco and its derivatives disrupt the calcium-phosphate balance required for bone matrix mineralization by inhibiting vitamin D and calcium absorption and affecting bone mineral density [20]. In their study evaluating the incidence of osteoporosis, Hijazi et al. [21] demonstrated that the incidence differs between smokers and nonsmokers. Furthermore, smoking increases chronic oxidative stress in the body, which affects bone metabolism and leads to a decrease in bone mineral density [22]. In conclusion, in our study, fractal and radiomorphometric analysis of panoramic images of individuals who use MP and smoke is a suitable method for examining changes in jawbone architecture.

There are limited studies in the literature examining the relationship between bone mineral density and smoking [23–25]. Basavarajappa et al. [25] found that FD measurements taken only from the anterior part of the mental foramen on digital panoramic radiography in male smokers were lower in tobacco (ST) and smokeless tobacco (SLT) users compared to controls. Furthermore, SLT users had lower FD values ​​compared to ST users. Santolia et al. [23] evaluated patients with oral lesions due to areca nut and tobacco use, demonstrating that FD values ​​in three different regions varied between groups. In this study, no significant differences were found in terms of FD between the three groups when examining the FA measurements in ROI 1, ROI 2, ROI 3, the mean ROI, and histogram values. The results of the studies are affected by methodological differences such as ROI selection and sizes, the superimposition of surrounding anatomical structures on the examined areas, anatomical differences between individuals and different demographic characteristics such as patient gender, number and age range, smoking frequency and type of cigarette used.

Some studies examining FD values ​​in various diseases have not identified a gender-related correlation [9]. The study population in this study consisted of men. Due to gender-related characteristics and hormonal factors that may influence the results, larger studies with different age and gender samples are needed to confirm these results. The strengths of this study include the use of FA, a non-invasive and quantitative method for evaluating trabecular bone structure, and the inclusion of numerous radiomorphometric parameters. Furthermore, although all measurements were performed by a single experienced observer, which may limit generalizability, the excellent intra-observer reliability supports the reproducibility of the measurement protocol. However, some limitations must be acknowledged. The relatively small sample size may have limited the statistical power to detect subtle differences. The retrospective design and reliance on panoramic radiographs also limit the generalizability of the findings. Additionally, the absence of clinical bone density measurements or biochemical markers of bone metabolism limits the ability to establish clinical correlations.

## Conclusion

Although no statistically significant differences were observed among MP users, smokers, and healthy controls in terms of fractal, radiomorphometric, or histogram-based parameters, the observed downward trend in fractal dimension suggests a potential early effect of MP on mandibular bone microarchitecture. Longitudinal studies with larger sample sizes and three-dimensional imaging modalities are warranted to further elucidate the potential impact of MP on bone quality and density.

## Data Availability

The data that support the findings of this study are available from the corresponding author upon reasonable request.
